# The Fruit Hull of* Gleditsia sinensis* Enhances the Anti-Tumor Effect of* cis*-Diammine Dichloridoplatinum II (Cisplatin)

**DOI:** 10.1155/2016/7480971

**Published:** 2016-09-18

**Authors:** Kyun Ha Kim, Chang-Woo Han, Seong Hoon Yoon, Yun Seong Kim, Jong-In Kim, Myungsoo Joo, Jun-Yong Choi

**Affiliations:** ^1^School of Korean Medicine, Pusan National University, Yangsan 50621, Republic of Korea; ^2^Department of Internal Medicine, Korean Medicine Hospital of Pusan National University, Yangsan 50621, Republic of Korea; ^3^Lung Cancer Clinic, Pulmonary Medicine Center, Pusan National University Yangsan Hospital, Yangsan 50621, Republic of Korea; ^4^Department of Internal Medicine, School of Medicine, Pusan National University, Yangsan 50621, Republic of Korea; ^5^Department of Acupuncture and Moxibustion, College of Korean Medicine, Kyung Hee University, Seoul 02447, Republic of Korea

## Abstract

Lung cancer has substantial mortality worldwide, and chemotherapy is a routine regimen for the treatment of patients with lung cancer, despite undesirable effects such as drug resistance and chemotoxicity. Here, given a possible antitumor effect of the fruit hull of* Gleditsia sinensis* (FGS), we tested whether FGS enhances the effectiveness of* cis*-diammine dichloridoplatinum (II) (CDDP), a chemotherapeutic drug. We found that CDDP, when administered with FGS, significantly decreased the viability and increased the apoptosis and cell cycle arrest of Lewis lung carcinoma (LLC) cells, which were associated with the increase of p21 and decreases of cyclin D1 and CDK4. Concordantly, when combined with FGS, CDDP significantly reduced the volume and weight of tumors derived from LLC subcutaneously injected into C57BL/6 mice, with concomitant increases of phosphor-p53 and p21 in tumor tissue. Together, these results show that FGS could enhance the antitumor activity of CDDP, suggesting that FGS can be used as a complementary measure to enhance the efficacy of a chemotherapeutic agent such as CDDP.

## 1. Introduction

Lung cancer is a malignant tumor with poor prognosis. The morbidity and mortality of lung cancer have increased annually. Approximately, 1.82 million people were diagnosed with lung cancer worldwide in 2012, which accounted for 13% of all cancers [1–3]. In Korea, the 73,759 cancer deaths were reported in 2012, of which 16,654 cases were due to lung cancer [4]. Lung cancer is typically treated by radiotherapy, chemotherapy, and surgical therapy [5]. However, chemotherapy remains as a major option for the treatment of lung cancer patients, although chemotherapeutic drugs accompany serious side effects, such as chemotoxicity and drug resistance [6]. Therefore, the substantial research effort in lung cancer therapy has focused on improving the efficacy and decreasing the adverse effects of chemotherapeutics by combining the conventional chemotherapy with complementary or alternative treatments such as herbal medicine [7].

In traditional Korean medicine, the fruit hull of* Gleditsia sinensis* (FGS) LAM (Leguminosae) has been used to treat various respiratory symptoms and subcutaneous pyogenic infections [8]. In mouse models, FGS suppresses lung inflammation in an LPS-induced acute lung injury [9, 10]. In addition to these, numerous experimental evidences suggest that FGS has antitumor activity without significant adverse effects. For instance, the ethanol extract of* G. sinensis *and its constituent saponin were reported to induce cancer cell apoptosis and to inhibit proliferation of various cancer cells, including breast cancer [11–13], colon cancer [14, 15], gastric cancer [16], esophageal cancer [11, 17, 18], liver cancer [11, 12], metastatic lung cancer [19], and leukemia [20, 21]. Given these experimental findings, in this study, we examined whether FGS enhances the antitumor effect of* cis*-diammine dichloridoplatinum II (CDDP), a chemotherapeutic drug that is frequently used to treat lung cancer patients, by using a lung cancer cell line, Lewis lung carcinoma (LLC), and a murine cancer model. Our results show that FGS could enhance the antitumor effect of CDDP by increasing the apoptosis and cell cycle arrest of LLC, suggesting a possible usage of FGS as a complementary or supplementary regimen to increase the efficacy of CDDP in cancer therapy.

## 2. Materials and Methods

### 2.1. Preparation of the Water Extract of* G. sinensis* Fruit Hull

The fruit of* G. sinensis *LAM (Leguminosae) was purchased from Kwang-Myoung-Dang Herb Store (Ulsan, Republic of Korea) and authenticated by Professor Chang-Woo Han at the School of Korean Medicine, Pusan National University (Yangsan, Republic of Korea). A voucher specimen (number: pnukh001) is kept in the School of Korean Medicine, Pusan National University. A decoction was prepared by boiling 300 g of the fruit hulls of* G. sinensis *in distilled water for two hours followed by filtration through a 0.45 *μ*m filter. The resultant decoction underwent a freeze-drying process to yield 60 g of powder (20% yield). An appropriate amount of the powder was dissolved in phosphate-buffered saline (PBS) prior to experimentation. The constituents of FGS were fingerprinted, as published previously [10].

### 2.2. Reagents

Anti-Bcl-2, caspase 3, caspase 7, cyclin B1, cyclin D1, CDK2, CDK4, CDC2, p21, p27, and *β*-actin antibodies were purchased from Santa Cruz Biotechnology (Santa Cruz, CA, USA), and anti-PARP and phospho-p53 (Ser 15) antibodies were from Cell Signaling Technology (Danvers, MA, USA). 3-(4,5-Dimethylthiazol-2-yl)-2,5-diphenyltetrazolium bromide (MTT) and propidium iodide (PI) were obtained from Sigma-Aldrich (St. Louis, MO, USA). Cisplatin (*cis*-diammine dichloroplatinum II; CDDP) was purchased from JW Pharmaceutical Co. (Seoul, Republic of Korea).

### 2.3. Cell Line and Culture Condition

Lewis lung carcinoma (LLC) cells derived from a C57BL/6 mouse were purchased from the American Type Culture Collection (Cat#: CRL-1642, Rockville, MD, USA) and cultured in DMEM medium (Gibco, Grand Island, NY, USA) supplemented with 10% fetal bovine serum (FBS), 100 *μ*g/mL penicillin, and 100 *μ*g/mL streptomycin at 37°C in a humidified atmosphere containing 5% CO_2_.

### 2.4. Cell Viability Assay

Cell viability was measured by 3-(4,5-dimethylthiazol-2-yl)-2,5-diphenyltetrazolium bromide (MTT) reduction assay. Cells were treated with CDDP (1, 3, 5, or 10 *μ*g/mL), FGS (50 *μ*g/mL), or CDDP 1 h prior to FGS treatment (CDDP 1 *μ*g + FGS 50 *μ*g, CDDP 3 *μ*g + FGS 50 *μ*g, CDDP 5 *μ*g + FGS 50 *μ*g, and CDDP 10 *μ*g + FGS 50 *μ*g). After the culture media were removed at 24 h after CDDP treatment, MTT solution was added to the cells, which were incubated for 4 h at 37°C. Formazan crystals formed in the viable cells were solubilized with dimethyl sulfoxide (DMSO), and the absorbance at 540 nm was determined by a spectrometer. The percentage of living cells was calculated against untreated cells.

### 2.5. Cell Cycle Analysis

Cells were treated with CDDP (1 or 3 *μ*g/mL), FGS (50 *μ*g/mL), or CDDP (1 or 3 *μ*g/mL) 1 h prior to 50 *μ*g/mL of FGS treatment. At 24 h after CDDP treatment, cells were washed with ice-cold PBS, fixed with 70% ice-cold ethanol, and suspended in propidium iodide (PI)/RNase A solution. Deoxyribonucleic acid content was analyzed by flow cytometry (FACS Canto II, Becton Dickinson, Franklin Lakes, NJ, USA).

### 2.6. Cell Apoptosis Analysis

Apoptosis was determined by an annexin V-FITC/PI double staining assay. After treatment with CDDP or FGS for 24 h as described above, cells were collected, washed with ice-cold PBS, and then stained with a solution containing annexin V-FITC and PI for 15 min in the dark at room temperature. The fluorescent signals in the cells were analyzed by the flow cytometry. After cell debris, characterized by a low forward/side scatter, was excluded, annexin V-positive cells in UR (upper right) and LR (lower right) were counted as apoptotic cells.

### 2.7. Animal Studies

Six-week-old male C57BL/6 mice were purchased from Samtaco Bio Korea, Ltd. (Osan, Republic of Korea). Animals were housed in certified, standard laboratory cages and were given food and water* ad libitum* prior to the experiment. All experimental procedures followed the guideline of the NIH of Korea for the Care and Use of Laboratory Animals, and all the experiments were approved by the Institutional Animal Care and Use Committee of Pusan National University. The duration of the experiment with mice was 3 weeks. In the first week, tumor-bearing mice were generated. C57BL/6 mice were injected subcutaneously with LLC cells (5 × 10^5^ cells in 50 *μ*L PBS) in the right flank. Seven days later, when the tumors were palpable, the mice were randomly divided into 4 groups (*n* = 10/group). From day 8, the tumor-bearing mice received NS or FGS (6.6 mg/kg body weight, equivalent to two doses prescribed for patients in Korean medicinal clinic in Korea) via gavage every day, with or without a single intraperitoneal (i.p.) injection of CDDP (3 mg/kg body weight) twice a week for the next 2 weeks. Mice were sacrificed at 21 days after the subcutaneous injection of LLC. The longest and perpendicular diameters of the tumor were measured by a caliper every three or four days. Tumor volume (Tv) was calculated by the formula Tv = 0.52 × *a* × *b* ^2^ (*a* is the largest superficial diameter, and *b* is the smallest superficial diameter).

### 2.8. Western Blot Analysis

Tumor tissue was frozen in liquid nitrogen and ground by milling in mortar. LLC cells and cells isolated from tumor tissue were lysed by RIPA buffer with protease inhibitor cocktail and the instruction from the manufacturer (Thermo Scientific, IL, USA). Proteins were then separated on 8–12% reducing SDS-PAGE gels and transferred onto nitrocellulose membranes (Bio-Rad Laboratories, Hercules, CA, USA) in 20% methanol, 25 mM Tris, and 192 mM glycine. Membranes were blocked with 5% nonfat dry milk and incubated overnight with primary antibodies at 4°C and subsequently with horseradish-peroxidase conjugated secondary antibody. The proteins of interest were developed with an enhanced chemiluminescence system (SuperSignal® West Femto, Thermo). Relative expression of each protein was shown over *β*-actin after the intensity of each band was determined by using the densitometric analysis software Image J (NIH; Bethesda, MD, USA).

### 2.9. Statistical Analysis

Data are presented as the mean ± SEM (standard error of the mean) from at least three separate experiments. For comparison among groups, one-way analysis of variance (ANOVA) tests with Tukey's post hoc test were used (with the assistance of InStat, Graphpad Software, Inc., San Diego, CA, USA).* P* value less than 0.05 was considered statistically significant.

## 3. Results

### 3.1. FGS Enhances the Proapoptotic Effect of CDDP on Lung Cancer Cell

**Figure 1:**
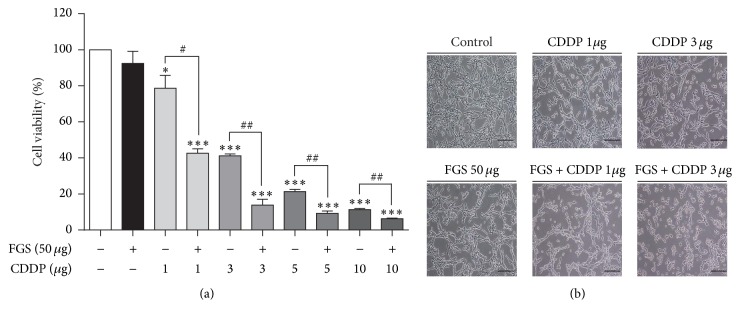
Effect of FGS on the viability of CDDP-treated LLC. LLC cells were treated with CDDP (1, 3, 5, or 10 *μ*g/mL), without or with FGS (50 *μ*g/mL). (a) Cell viability was measured by MTT assay 24 h after treatment. Each column represents the mean ± SEM of three measurements (^*∗*^ *P* < 0.05 and ^*∗∗∗*^ *P* < 0.005, compared with untreated control; ^#^ *P* < 0.05 and ^##^ *P* < 0.01, compared with the FGS-treated group). (b) After cells were treated as in (a), cell morphology was examined under the microscope (magnification: 100x; scale bar = 50 *μ*m). Shown are representatives of 5 different microscopic fields of each sample.

To test whether FGS enhances the effect of CDDP, we first determined whether FGS influences the effect of CDDP on cell viability. LLC cells were treated with increasing amounts of CDDP (1, 3, 5, and 10 *μ*g/mL), without or with 50 *μ*g/mL of FGS administered 1 h later. At 24 h after CDDP treatment, cells were harvested for MTT assay. As shown in Figure 1(a), while FGS (50 *μ*g/mL) alone did not significantly affect the viability of LLC cells (1st and 2nd columns), CDDP decreased the viability of LLC cells in a dose-dependent fashion (3rd, 5th, 7th, and 9th columns), compared with the untreated control. When combined with FGS, CDDP significantly decreased the cell viability further, compared with the CDDP-treated cells (4th, 6th, 8th, and 10th columns). Similarly, FGS enhanced the morphologic shrinkage of the cells induced by CDDP (Figure 1(b)). These results suggest that FGS enhances the effect of CDDP on cell viability.

**Figure 2:**
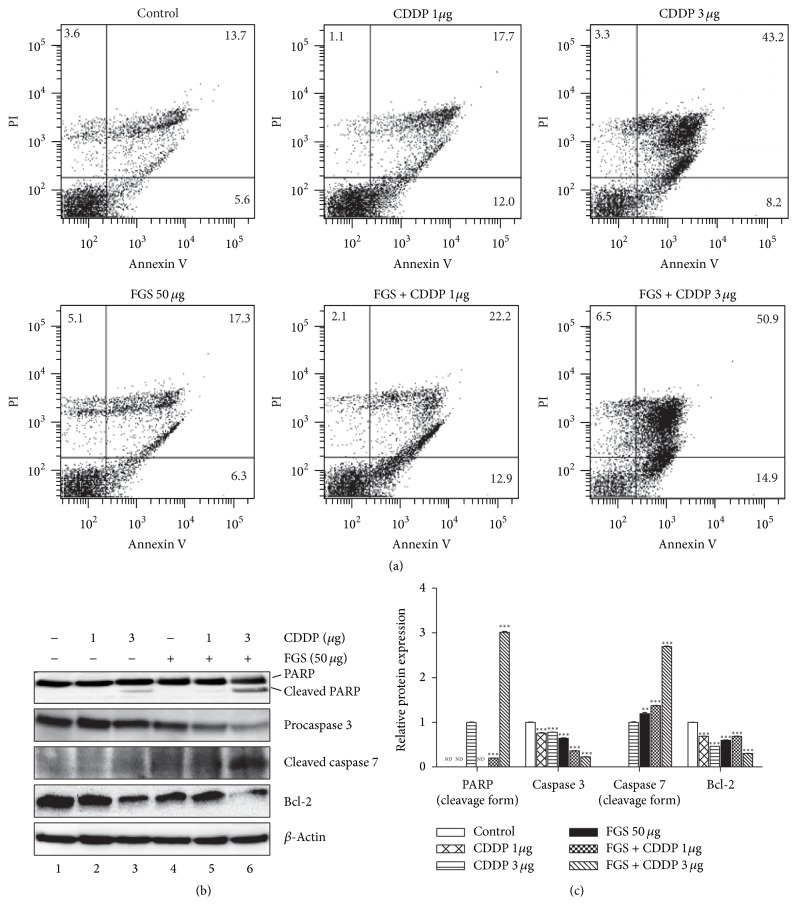
Effect of FGS on the apoptosis of CDDP-treated LLC. After treatment with CDDP (1 or 3 *μ*g/mL), without or with FGS (50 *μ*g/mL), LLC cells were harvested for annexin V/PI double staining assay (a). The percentages of negative cells (viable cells), annexin V-positive cells (apoptotic cells), PI-positive cells (necrotic cells), or annexin V- and PI-positive cells (late apoptotic cells) were measured by FACS analysis. (b) Total proteins in the cells treated as in (a) were analyzed by western blotting for apoptotic factors. Each band was quantitated by a densitometer, and relative expression of each protein was shown over *β*-actin (c). Each column represents the mean ± SEM of three measurements (ND: none detected; ^*∗∗*^ *P* < 0.01 and ^*∗∗∗*^ *P* < 0.005, compared with untreated control).

Next, we examined whether FGS enhances the proapoptotic effect of CDDP [22, 23]. Similar to Figure 1, LLC cells were treated with CDDP alone, or along with FGS, and the apoptosis of LLC was determined by annexin V-FITC/PI double staining assay. As shown in Figure 2(a), CDDP alone increased the apoptosis of LLC cells from 19.3% (control) to 29.7% (CDDP 1 *μ*g/mL) or to 51.4% (CDDP 3 *μ*g/mL), while FGS alone marginally increased the apoptosis of LLC from 19.3% to 23.6%. However, when combined with CDDP, FGS enhanced the apoptosis elicited by CDDP from 29.7% (CDDP 1 *μ*g) to 35.1% (FGS + CDDP 1 *μ*g) and from 51.4% (CDDP 3 *μ*g) to 65.8% (FGS + CDDP 3 *μ*g). To examine whether FGS increasing apoptosis is associated with activation of the factors that are involved in cell apoptosis, such as PARP, caspase 3, caspase 7, and Bcl-2, we performed western blotting for the factors. As shown in Figures 2(b) and 2(c), CDDP increased the levels of cleaved PARP and cleaved caspase 7 and decreased those of procaspase 3 and Bcl-2 (lanes 2 and 3), while FGS alone did the same, albeit to a lesser degree (lane 4). However, combined with FGS, CDDP significantly increased the levels of cleaved PARP and cleaved caspase 7 and decreased those of procaspase 3 and Bcl-2 (lane 5), which were more robust with a high dose of CDDP (lane 6). Together, these results suggest that FGS significantly enhances the proapoptotic activity exerted by CDDP.

### 3.2. FGS Enhances the Effect of CDDP on Cell Cycle Arrest

**Figure 3:**
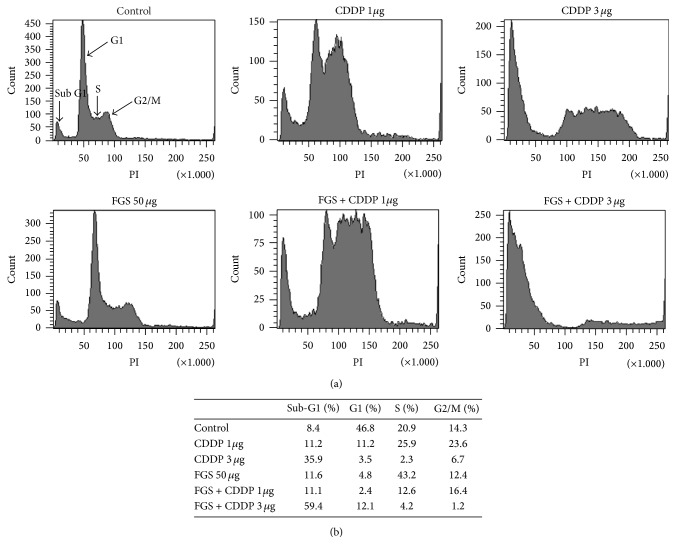
Effect of FGS on the cell cycle progression of CDDP-treated LLC. LLC cells were treated with CDDP (1 or 3 *μ*g/mL), without or with FGS (50 *μ*g/mL). At 24 h after treatment, cells were harvested, stained with PI, and analyzed by FACS (a). The percentages of cells in each cell cycle stage are shown in (b).

Since CDDP blocks the progress of cell cycle [22, 23], we examined whether FGS enhances the suppressive effect of CDDP on cell cycle. LLC was treated with CDDP and FGS as described above, and different cell populations of LLC based on DNA content were determined by FACS analysis. As shown in Figure 3, untreated LLC cells (control) could be categorized into four different populations: sub-G1, G1, S, and G2/M [24]. While CDDP at the low dose (1 *μ*g/mL) decreased the LLC population at G1 stage (46.8% to 11.2%) and increased the populations at sub-G1 (8.4% to 11.2%), S (20.9% to 25.9%), and G2/M stages (14.3% to 23.6%), CDDP at the high dose (3 *μ*g/mL) increased the LLC population mostly at sub-G1 stage (8.4% to 35.9%), suggesting that CDDP inhibits the cell cycle transition through G1 stage. On the other hand, while FGS stalled LLC cells largely at S stage, FGS combined with CDDP (3 *μ*g/mL) arrested LLC largely at sub-G1 stage, the population at which was higher than CDDP (3 *μ*g/mL) alone (35.9% versus 59.4%). These results suggest that FGS helps enhance the activity of CDDP in suppressing the transition through G1.

**Figure 4:**
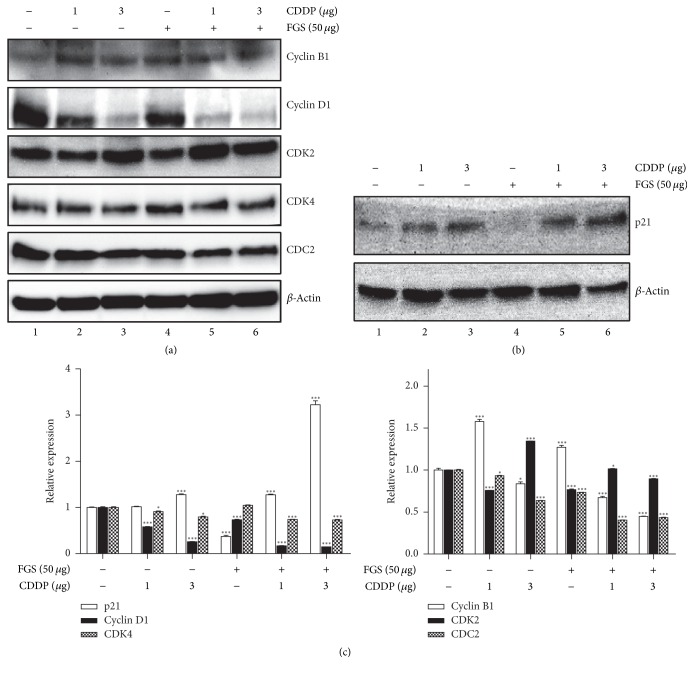
Effect of FGS on the factors that regulate the cell cycle of CDDP-treated LLC. LLC cells, treated with CDDP (1 or 3 *μ*g/mL), without or with FGS (50 *μ*g/mL), were analyzed by western blotting for cyclins and cyclin-dependent kinases (a) and for p21 (b). Each band was analyzed by a densitometer, and the relative expression of each protein was shown over *β*-actin (c). Each column represents the mean ± SEM of three measurements (^*∗*^ *P* < 0.05 and ^*∗∗∗*^ *P* < 0.005, compared with untreated controls).

To examine whether FGS, along with CDDP, blocks the G1 transition, we performed western blotting for the factors that regulate cell cycle progression through G1, including cyclin D1 and cyclin-dependent kinases (CDKs) 2 and 4. As shown in Figure 4(a), while FGS did not affect the expression of these proteins (lane 4), CDDP robustly reduced the expression of cyclin D1 (lanes 2 and 3), which was further decreased by FGS (lanes 5 and 6). Since the cell cycle transition through the G1 stage is inhibited by p21, we similarly measured the level of p21 in LLC that was treated with CDDP and FGS. As shown in Figure 4(b), CDDP increased the expression of p21 in LLC cells (lanes 2 and 3), which was enhanced by FGS (lanes 5 and 6). Densitometric analysis of these proteins reveals that FGS significantly enhanced the effects of CDDP on the expression levels of the factors that regulate the G1 transition (Figure 4(c)). Together, these results suggest that FGS enhances the function of CDDP in suppressing the G1 transition of LLC cells, resulting in accumulated population at sub-G1 stage.

### 3.3. FGS Enhances the Effect of CDDP on Tumor Growth in Mice

**Figure 5:**
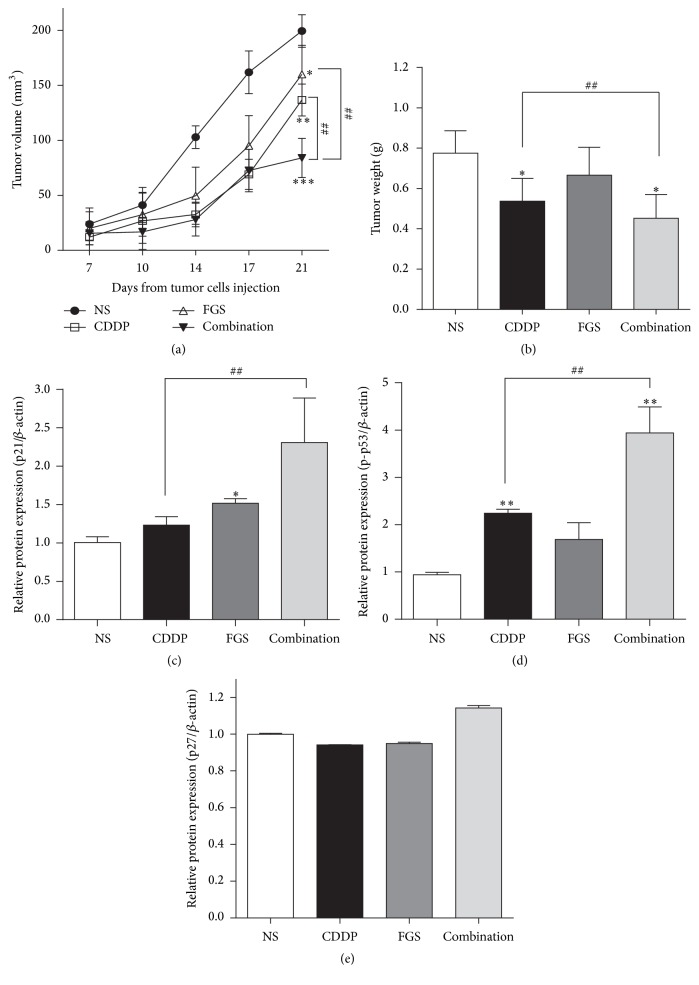
FGS, combined with CDDP, suppresses LLC-derived tumor in mice. Male mice (*n* = 10/group) received subcutaneous LLC injection and 7 days later were treated with sham (normal saline: NS), FGS, or CDDP (3 mg/kg) without or with FGS for indicated periods. The volume (a) and weight (b) of the tumor were measured every other day (^*∗*^ *P* < 0.05, ^*∗∗*^ *P* < 0.01, and ^*∗∗∗*^ *P* < 0.005, compared with the NS-treated group; ^##^ *P* < 0.01, compared with CDDP- or FGS-treated group). The tumor was surgically removed and analyzed by western blotting for p21 (c), phospho-p53 (d), and p27 (e). Each band was quantitated by a densitometer and relative expression of each protein was calculated over *β*-actin. Each column represents the mean ± SEM of three measurements (^*∗*^ *P* < 0.05 and ^*∗∗*^ *P* < 0.01, compared with the NS-treated group; ^##^ *P* < 0.01, compared with the CDDP-treated group).

Because FGS, combined with CDDP, enhanced the suppressive effects of CDDP on LLC cell growth, we tested whether FGS does similarly against tumor growth in mice. C57BL/6 mice were subcutaneously injected with LLC cells. At day 7 after the injection, when tumor growth was detectable, the mice received oral administration of FGS, i.p. CDDP, or both oral FGS and i.p. CDDP. The effect of these differential treatments on tumor growth was monitored for 2 weeks. As shown in Figures 5(a) and 5(b), CDDP treatment significantly decreased the volume (31.43%) and weight (39.18%) of LLC-derived tumor, compared with untreated controls. While the effect of FGS was marginal, CDDP combined with FGS significantly decreased the volume (57.89%) and weight (48.79%) of the tumor, suggesting that FGS enhances the suppressive effect of CDDP on the tumor growth in mice. It is of note that mice did not show any sign of weight loss or compromised activity during the experiment (data not shown). In order to determine whether tumor growth suppressed by FGS and CDDP is related to cell cycle arrest, as found in LLC cells, we measured the level of p21 in the tumor tissue by western blot analysis. As shown in Figure 5(c), CDDP combined with FGS significantly increased the level of p21, compared with CDDP only. Consistent with this, CDDP combined with FGS enhanced the phosphorylation of p53 (Figure 5(d)), indicative of activated p53, which induces the expression of p21 [25]. On the other hand, FGS, CDDP, or both FGS and CDDP did not significantly affect the expression of p27, a homolog of p21 that regulates the G1 transition [26] (Figure 5(e)). Together, these results suggest that FGS helps enhance the antitumor effect of CDDP, which is associated with enhanced cell cycle arrest of tumor in mice.

## 4. Discussion

CDDP is a representative chemodrug used for the treatment of cancer patients [22, 23]. However, CDDP frequently comes with drug resistance and serious side effects [6]. To circumvent these adversaries, combined modality therapies have been explored, where chemotherapeutic agents are administered along with other drugs or natural herbal medicinal products [27]. In this study, given the anticancer properties of FGS [11–21], we set up and tested a hypothesis that FGS enhances the anticancer effect of CDDP by using LLC cell and a murine lung cancer model. We found that, with a dose showing a minimal anticancer effect, FGS enhanced significantly the antitumor effect of CDDP. Analyses of proteins in LLC cells and LLC-derived tumor tissue in mice reveal that FGS significantly enhanced the effects of CDDP in promoting apoptosis and prohibiting cell cycle transition through the G1 stage. Therefore, our findings suggest that FGS can be used as a complementary regimen to improve the efficacy of chemotherapy with CDDP.

The anticancer effects of chemotherapeutic drugs are associated with increased apoptosis and cell cycle arrest [28]. Therefore, we first examined whether FGS can enhance the proapoptotic effect of CDDP on a lung cancer cell line, LLC. Our results show that while 50 *μ*g/mL of FGS increased apoptosis marginally, the same amount of FGS significantly enhanced the cell apoptosis elicited by CDDP, as determined by FACS analysis of annexin V-positive cell population. In support of these findings, FGS enhanced the effects of CDDP on the activation of proteins involved in the regulation of apoptosis, such as PARP, procaspase 3, and procaspase 7, with a concomitant decrease of antiapoptotic factor Bcl-2. Next, we examined whether FGS enhances the function of CDDP in regulating the cell cycle. While CDDP at a low dose increased the cell populations at S and G2/M stages, CDDP at a higher dose constellated the cells mostly at sub-G1 stage. With CDDP, FGS robustly increased the population at sub-G1 stage, suggesting that cotreatment with FGS and CDDP affects the G1 transition. Since the G1 transition is promoted by cyclin D1 that forms a complex with CDK4 or CDK6 [24, 29] and blocked by p21, a Cip/Kip family protein that inhibits the activity of CDKs [30], we measured them by western blot analysis. Our result shows that FGS with CDDP strongly decreased the level of cyclin D1 and increased the expression of p21, suggesting that FGS combined with CDDP prevents the G1 transition through suppression of cyclin D1 and activation of p21. Although FGS at the dose used in the study increased the cell populations at sub-G1 and S stages, our finding that FGS facilitated the suppression of the G1 transition is consistent with an early finding that the ethanol extract of FGS arrests cell cycle at G0/G1 phase in gastric cancer cell [16].

The role of FGS in enhancing the anticancer effect of CDDP was further tested in a murine cancer model. Since cancerous cells are, in principle, syngeneic to the host, we examined the effect of FGS on CDDP by using a syngeneic mouse model, in which LLC cells, whose origin is C57BL/6 mice [31], were injected into C57BL/6 mice. We presumed that LLC cells generate tumor tissue without eliciting a significant immune response from C57BL/6 mice. Concordantly, we could generate tumor tissue in C57BL/6 with no difficulty. According to a study with C57BL/6 mice, when administered at 5 mg/kg body weight, CDDP generates the toxicities in kidney and liver [32]. Therefore, we chose 3 mg/kg body weight of CDDP administered to C57BL/6 mice, which we considered suboptimal in eliciting the toxicities. There was no apparent change observed in the weights of the kidney and the liver, compared to sham-treated mice (data not shown). In addition, we did not encounter a premature death of mice during the experiment. However, we could not exclude the possibility that 3 mg/kg of CDDP gives toxicity to those organs because we did not specifically measure the markers that indicate the toxicity to the kidney and the liver in mice. Nevertheless, when administered with 3 mg/kg of CDDP, FGS significantly lowered the volume and the size of the tumor in mice. Molecular analyses show that, consistent with the results with LLC, expressions of p21 and p53 were elevated in the LLC-derived tumor. It is of note that CDDP activates p53 [33], which increases the expression of p21, blocking the G1 transition [34, 35]. Given that cyclin D1 overexpression has been shown to correlate with early cancer onset and tumor progression [34], FGS can be used broadly to enhance the efficacy of a chemotherapeutic drug such as CDDP in treating various cancer. It is of note that mice, in this study, received only two-thirds of a daily dose prescribed to patients in Korean medicinal clinic. Therefore, it is possible that as the amount of FGS administered to mice increases, the anticancer function of CDDP is enhanced to a degree. Nonetheless, our results strongly suggest that FGS enhances the anticancer activity of CDDP.

CDDP has been subjected to studies for combinational therapy, because of CDDP-resistance [6] and of accompanying side effects, including nephrotoxicity [36], neurotoxicity [37], and ototoxicity [38]. The purpose of this study was to address whether a limited amount of FGS enhances the anticancer effect of CDDP, in the hope of contributing to decreasing the CDDP-resistance. Given FGS enhancing the anticancer effect of CDDP, it would be also interesting to examine whether FGS alleviate the toxicity to kidney and liver incurred by CDDP.

## 5. Conclusions

CDDP, combined with FGS, exhibited enhanced antitumor effects in a murine lung cancer cell line and a tumor-bearing murine model. FGS enhanced the functions of CDDP in cell apoptosis and in blocking the G1 transition, which was associated with decreased cyclin D1 and increased p21 in LLC and LLC-derived tumor tissue. Given the implication of cyclin D and p21 in numerous cancers, our results suggest that FGS can be used to improve the effectiveness of various chemotherapeutics including CDDP.
